# Direct Interaction of Endogenous Kv Channels with Syntaxin Enhances Exocytosis by Neuroendocrine Cells

**DOI:** 10.1371/journal.pone.0001381

**Published:** 2008-01-02

**Authors:** Dafna Singer-Lahat, Dodo Chikvashvili, Ilana Lotan

**Affiliations:** Department of Physiology and Pharmacology, Sackler Faculty of Medicine, Tel-Aviv University, Ramat-Aviv, Israel; Emory University, United States of America

## Abstract

K^+^ efflux through voltage-gated K^+^ (Kv) channels can attenuate the release of neurotransmitters, neuropeptides and hormones by hyperpolarizing the membrane potential and attenuating Ca^2+^ influx. Notably, direct interaction between Kv2.1 channels overexpressed in PC12 cells and syntaxin has recently been shown to facilitate dense core vesicle (DCV)-mediated release. Here, we focus on endogenous Kv2.1 channels and show that disruption of their interaction with native syntaxin after ATP-dependent priming of the vesicles by Kv2.1 syntaxin–binding peptides inhibits Ca^2+^ -triggered exocytosis of DCVs from cracked PC12 cells in a specific and dose-dependent manner. The inhibition cannot simply be explained by the impairment of the interaction of syntaxin with its SNARE cognates. Thus, direct association between endogenous Kv2.1 and syntaxin enhances exocytosis and in combination with the Kv2.1 inhibitory effect to hyperpolarize the membrane potential, could contribute to the known activity dependence of DCV release in neuroendocrine cells and in dendrites where Kv2.1 commonly expresses and influences release.

## Introduction

Many proteins have been identified as essential to the exocytosis process including the proteins of the soluble N-ethylmaleimide-sensitive factor attachment protein receptors (SNARE); syntaxin 1A, SNAP-25 and the vesicle membrane associated VAMP 2 (synaptobrevin) [1]–[6]. Other proteins have been implicated in the fusion complex of regulated exocytosis including voltage-gated Ca^2+^ channels N-, P/Q and L-type [7]–[10]; for reviews see [11] and [12].

Voltage gated K^+^ (Kv) channels can indirectly inhibit the secretion of neurotransmitters from presynaptic terminals [13], [14], and neuropeptides and hormones from neuroendocrine and endocrine cells [15] by limiting the Ca^2+^ influx through voltage-gated Ca^2+^ channels, due to outward K^+^ currents through their pore that shape membrane depolarization. Noting that Kv2.1 channels interact directly with syntaxin 1A and with SNAP-25 in PC12 cells, oocytes and islet β-cells [15]–[20], overexpression of Kv2.1 was shown to enhance the release of pro-ANF (atrial natriuretic factor) by living PC12 cells, independent of the channel's pore function. In addition, syntaxin-binding peptides, derived from cytoplasmic portions of the channel, decreased syntaxin coimmunoprecipitation with Kv2.1 and inhibited the Kv2.1-dependent facilitation of release [21]. Thus, we postulated that direct association of overexpressed Kv2.1 with syntaxin promotes exocytosis. Furthermore, expression of a mutant form of the channel that lacks the syntaxin-binding C1a domain (Kv2.1ΔC1a) not only failed to enhance release, as expected, but also slightly inhibited it. The inhibition was attributed to the co-assembly of Kv2.1ΔC1a and wild-type endogenous Kv2.1 subunits to form heterotetramers with a reduced syntaxin-binding capacity, implying that the interaction of endogenous Kv2.1 channels with syntaxin may play a role in the release of DCVs by PC12 cells.

Here, we examined this notion in cracked PC12 cells that undergo MgATP priming and Ca^2+^ triggering to release norepinephrine (NE) stored in DCVs [22]. We show that impairment of the interaction of syntaxin with endogenous Kv2.1 attenuates NE release. Moreover, we show that impairment of the syntaxin-Kv2.1 interaction during the triggering phase is responsible for the reduced release. Thus, in neuroendocrine cells endogenous Kv channels enhance release by binding a key component of the fusion machinery.

## Results

**Figure 1:**
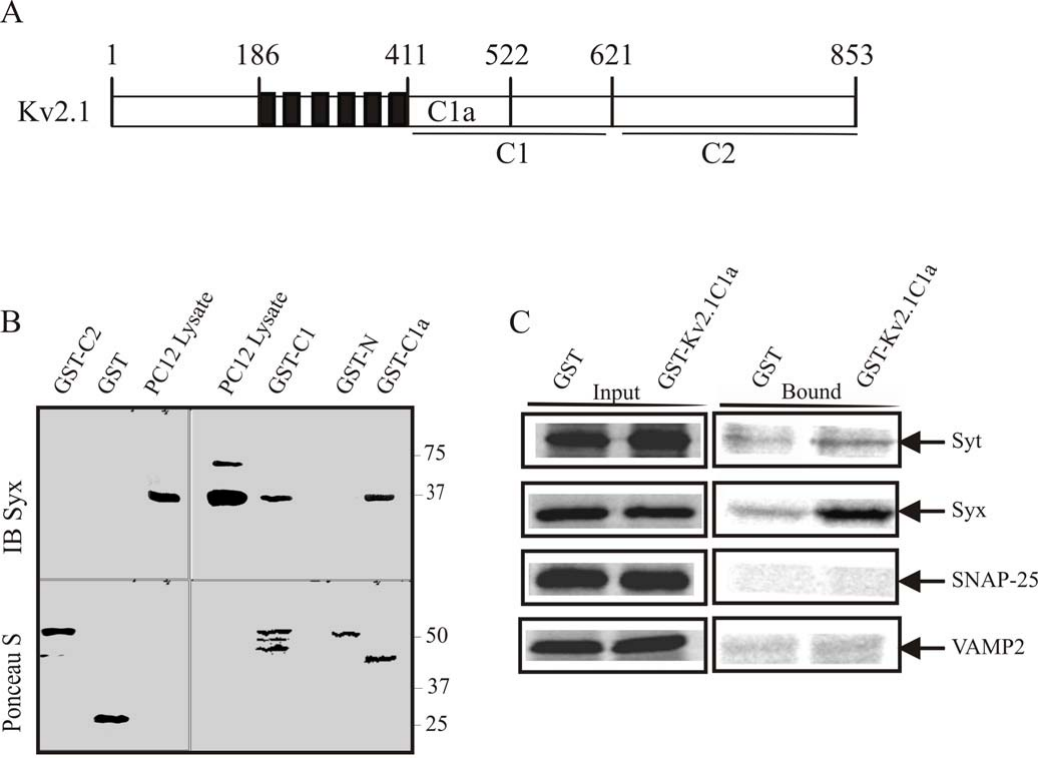
Recombinant peptides corresponding to Kv2.1 C terminus interact with native syntaxin in PC12 cells and in vitro, slightly with synaptotagmin but not with VAMP2 or SNAP-25 in vitro. A. A schematic presentation of the Kv2.1 channel showing fragments generated as GST fusion proteins. The grey box on the left, the 6 dark boxes and the grey box on the right denote the N terminus, the 6 trans membrane and the C terminus domains, respectively. Numbers denote amino acids. B. GST fusion proteins corresponding to Kv2.1 C terminus but not N terminus pull down syntaxin from PC12 cell lysate. Proteins (each at 150 pmol) immobilized on glutathione affinity beads were incubated with 10^6^ cells in K-Glu buffer with 1% Triton X-100 for 12 hr at 4°C. Precipitated proteins were separated by SDS-PAGE (12% polyacrylamide) and immunobloted with syntaxin antibody (*IB Syx*), or stained with Ponceau S (lower panel). PC12 lysates (0.5% of that processed in the pull down reaction) were loaded for reference. Right and left panels are two separate experiments. Molecular weights are marked on the right. C. GST-fused Kv2.1-C1a protein or GST alone (150 pmole each) immobilized on glutathione affinity beads were incubated with ^35^S-labeled synaptotagmin (*Syt*), ^35^S-labeled syntaxin (*Syx*), ^35^S-labeled SNAP-25 or ^35^S-labeled VAMP2 for 1 h. in 1 ml 0.1% Triton X-100. Gluthation-eluted proteins (*right panel*) and input (20 µl out of 1000 µl of reaction mixture taken before the addition of beads; *left panel*) were analyzed by SDS-PAGE.

Previous *in vitro* binding studies, using immobilized glutathione S-transferase (GST) fusion peptides corresponding to the major cytoplasmic parts of Kv2.1 (depicted in Fig. 1A), showed that syntaxin 1A (syntaxin) binds Kv2.1 C-terminal C1 and C1a domains, but not the Kv2.1 N terminus [23]. Here, we further characterized the binding of native syntaxin from PC12 cells to the Kv2.1 peptides. Syntaxin-immunoreactive bands were pulled down from PC12 cell lysates by Kv2.1-C1 and Kv2.1-C1a peptides (Fig. 1B), but not by the Kv2.1-N or Kv2.1–C2 peptides (the binding of syntaxin to the latter was very weak, if any). Concentrating on the Kv2.1-C1a peptide, we aimed at evaluating its profile of binding with the major protein components of the exocytotic machinery. Fig. 1C shows that under our binding conditions, the intensity of syntaxin binding was significantly larger than those of SNAP-25, VAMP2 or synaptotagmin, although a small binding of synaptotagmin was observed. It should be noted that in a different set of experiments, some VAMP2 binding to Kv2.1 C1a peptide was observed; its intensity was always significantly lower than that of syntaxin (not shown). We concluded that syntaxin was main Kv2.1-C1a binding protein.

**Figure 2:**
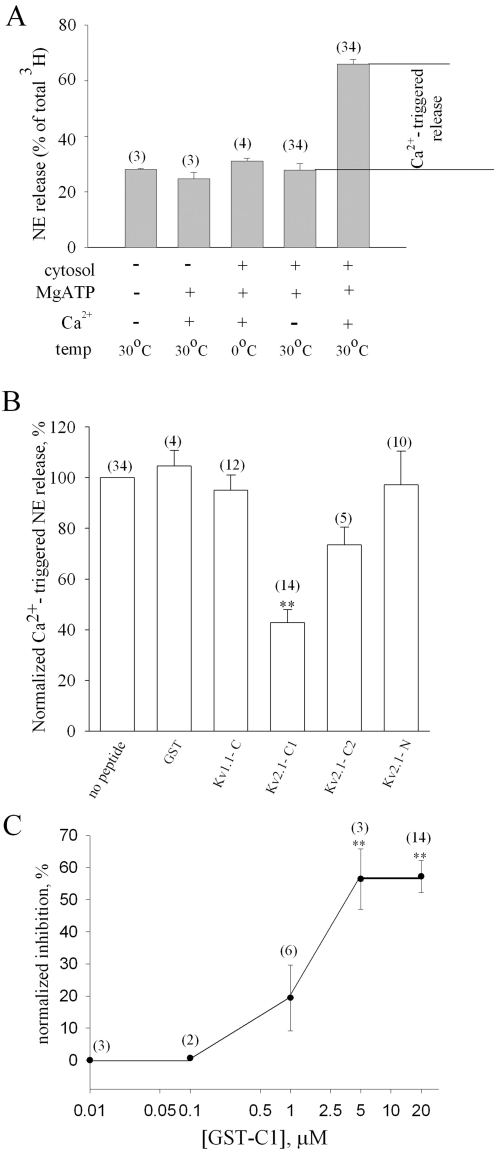
Kv2.1 syntaxin-binding peptides inhibit NE secretion. (A) PC12 cells preloaded with [^3^H]-NE and gently homogenized ( “cracked” cells), either underwent 15 min incubation in the presence of 2 mM MgATP and brain cytosol at 30°C (“priming”) or not, followed by either 15 min incubation with added 1.6 mM Ca^2+^ (1 µM free Ca^2+^ ;[39] at 30° (“triggering”) or not, as indicated below the bars. Cells were pelleted and the secreted [^3^H]-NE in the supernatant was quantified by scintillation counting and expressed as a percentage of the total [^3^H]-NE in the cell. 10^6^ cells per reaction were used. The difference between the amounts of [^3^H]-NE secreted in the two reactions on the two bars on the right was defined and is referred to hereafter as the Ca^2+^-triggered release. (B) Cracked cells preloaded with [^3^H]-NE underwent priming and triggering reactions in the absence or presence of 10–20 µM GST-fusion peptides corresponding to cytosolic parts of Kv2.1 and Kv1.1 (shown in Fig. 1A) or to GST itself (as indicated below bars). Values of Ca^2+^-triggered release measured in the presence of the peptides (see Fig. 2A) were normalized to the control release determined in the absence of peptides (defined as 100%). Each bar in A and B depicts the mean±s.e.m from several independent experiments (numbers of experiments are in parentheses above bars). **, *p*<0.001 (compared with GST). (C) Cells were stimulated to release NE in the presence of increasing concentrations of GST-fused Kv2.1-C1 protein. Each point in the curve represents mean±s.e.m values from several independent experiments (numbers of experiments in parentheses above bars). **, p<0.001 (compared with 10 nM GST-C1).

Next, in an effort to evaluate the involvement of the interaction between endogenous Kv2.1 and syntaxin in the Ca^2+^-regulated secretion from PC12 cells, we aimed to disrupt this interaction and to assay the impact on secretion. The above Kv2.1-derived syntaxin-binding peptides were shown to compete for the *in situ* association of syntaxin with Kv2.1 in PC12 cells [21] and, hence, were suited for this purpose. We expected that, if indeed this interaction facilitates secretion, its impairment will inhibit secretion. First, we ascertained that the peptides did not impair significantly the interaction of syntaxin with its partners in the fusion machinery. Significant impairment would inhibit release by itself. Indeed, the Kv2.1-C1a did not interfere with the associations between syntaxin and SNAP-25 or VAMP2, the protein components of the heterotrimeric SNARE complex formation, and interfered only slightly with the association between syntaxin and synaptotagmin (as compared with the marked disruption of the association between syntaxin and Kv2.1[21]) (Fig S1 and Text S1). As a secretion assay we chose the reconstituted exocytosis system of cracked cells in which, although the cell membranes are mechanically disrupted, organelles, docked dense-core vesicles (DCVs) and cytoskeleton are retained, thereby allowing secretion to be induced [22], [24], [25]. The Ca^2+^ -dependent release of [^3^H]-NE loaded onto the cells requires 30°C, MgATP and cytosolic proteins to perform priming of docked DCVs, followed by Ca^2+^ ions to trigger fusion [22], [24]. The Ca^2+^ -dependent release is calculated by subtracting the Ca^2+^ -independent release from the total release, measured in cells that only underwent priming but not triggering reactions (Fig. 2A). The cracked cells allowed penetration of peptides which are ∼50 kDa and below (verified by successful penetration of various fluorescently labeled IgG molecules into the inner part of the cells; not shown) so that the Kv2.1-derived peptides could be introduced during the priming and triggering reactions. A peptide corresponding to the C-terminus of Kv1.1, Kv1.1-C, served as an absolute control as it does not bind any syntaxin under any experimental conditions [26]. Ca^2+^ -triggered release in the presence of a peptide was normalized to the release in its absence (Fig 2B, *no peptide*), measured in the same experiment. The syntaxin-binding peptide, Kv2.1-C1, blocked the Ca^2+^-dependent release by about 60%, whereas Kv2.1-N and the negative control Kv1.1-C, or GST itself, did not significantly affect the release (Fig. 2B). In the presence of Kv2.1-C2 that binds syntaxin weakly [15], [23] there was a relatively small and statistically insignificant reduction of release (Fig. 2B).

To further substantiate the inhibitory effect, release was assayed in the presence of different concentrations of the syntaxin-binding peptide Kv2.1-C1 (Fig. 2C). A dose-dependent inhibition of release was observed. Under our release conditions, inhibition was half maximal at ∼2 µM peptide and at a saturation concentration of the peptide of 5 µM, the maximal inhibition achieved was about 57%. Fitting the dose response curve with Hill equation yielded a coefficient of 2.1, indicating that the minimal apparent stoichiometry of the interaction between GST-C1 and the exocytotic machinery was 2∶1. To ensure that the lack of inhibition by the syntaxin-nonbinding peptide Kv1.1-C was not due to protein availability, we tested its effect at a very high concentration of 60 µM which inhibited secretion by 20% (not shown). This small inhibition was probably due to a non specific interference with protein-protein interactions. These confirmed that the inhibition by the syntaxin-binding peptide was specific.

**Figure 3:**
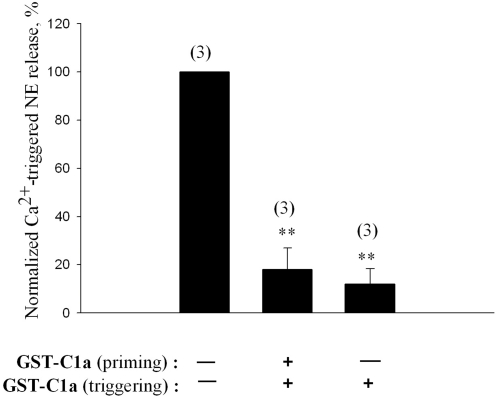
Inhibition of Ca^2+^-triggered release from cracked PC12 cells by a Kv2.1 syntaxin-binding peptide is conferred at the triggering step. Cells were stimulated to secrete [^3^H]-NE in the presence of GST-C1a, which was introduced during both the priming and the triggering phases (ATP and cytosol were present throughout (as in Fig. 2); middle bar) or during the triggering phase only (ATP was washed out before the triggering phase; right bar). Data shown are mean±s.e.m values from a single experiment performed in triplicate and normalized to the values determined in the absence of the peptide (left bar).

Next, we aimed to establish the stage at which the syntaxin-binding peptides exert their inhibition. We asked if the maximal inhibition of secretion by the peptides requires their presence during both the priming and triggering reactions (as was the case in Fig. 2B,C), or whether their presence during the triggering phase suffices. In these experiments we used the Kv2.1-C1a peptide which corresponds to the syntaxin binding domain shown to mediate the enhanced DCVs' release [21]. Two different experimental settings with the peptide were assayed, each compared to the assay in the absence of the peptide, in cells of a single batch. The first setting was performed as in Fig. 2B, namely, the peptide was introduced during the priming reaction together with MgATP and brain cytosol which remained throughout the triggering reaction during which Ca^2+^ was added. In the second setting already primed cells that were washed out of MgATP and brain cytosol were used and the peptide was introduced during the Ca^2+^ -triggering incubation together with cytosol but in the absence of MgATP. The latter was omitted from the triggering phase in order to exclude any continuation of the priming reaction. The results of the two settings were normalized to the control release measured as in the first setting but in the absence of peptide. In both experimental settings the inhibitory effect of Kv2.1-C1a was marked (Fig. 3). The greater effect apparent in the second setting was due to absence of MgATP (see Fig. S2 and Text S2). We concluded that the syntaxin-binding peptide, Kv2.1-C1a, interferes with the exocytic machinery during the triggering reaction.

## Discussion

This study is a direct continuation of and complement to our previous study [21]; both studies strongly suggest that Kv2.1-syntaxin interaction causes an enhancement of the release of DCVs. Whereas the previous study focused on overexpressed channels and showed that their interaction with endogenous syntaxin enhanced the release of green fluorescent protein-labeled pro-ANF, this study focused on endogenous channels and shows that impairment of their interaction with syntaxin significantly reduces [^3^H]-NE release. Thus, the results of this study importantly reveal that this phenomenon indeed occurs under physiological conditions in a neuroendocrine cell. Notably, in both studies, the impact of the Kv2.1-syntaxin interaction on secretion was studied under conditions in which K^+^ ion efflux and membrane potential changes were either minimal (secretion from living cells elicited by increasing the extracellular K^+^ concentration ; [21] or irrelevant (secretion from cells with permeable plasma membranes; this study). Thus, the impact of Kv2.1-syntaxin interaction does not result from a change in the voltage-dependent gating of the channel, but rather reflects the effect of a direct interaction between the channel and the secretory machinery

In this study syntaxin-binding peptides, Kv2.1-C1 and Kv2.1 C1a, were used to impair the interaction of endogenous Kv2.1 channels with syntaxin, probably with the open conformation of syntaxin which was shown to preferentially bind the channel [27]. We excluded the possibility that the binding of the peptides to syntaxin interfered significantly with its association with SNAP-25, VAMP2 or synaptotagmin which could result in inhibition of Ca^2+^-triggered secretion. However, we did not exclude the less likely possibility that the binding of other proteins to syntaxin, e.g., the binding of Munc 18 to assembled SANRE complexes containing syntaxin in the “open” conformation ([28] or Munc −13 which stabilizes the “open” conformation of syntaxin [29], [30], could be impaired and result in inhibition of Ca^2+^-triggered secretion.

Previously [21], we suggested several possible mechanisms that could be the basis of the facilitation of exocytosis by the association of syntaxin with the Kv channel. Firstly, in view of the proposal that syntaxin lines the fusion pore [31], the Kv2.1-syntaxin interaction may promote full collapse fusion, which induces complete release, in place of the incomplete release associated with kiss and run exocytosis. Secondly, in view of the demonstration that Kv2.1 preferentially binds the open conformation of syntaxin [27], the Kv2.1-syntaxin interaction may influence the equilibrium between the closed and open conformations of syntaxin. Thirdly, the Kv2.1-syntaxin interaction may influence the equilibrium between the reserve and the releasable pools of vesicles. In any of these or other possible mechanisms, both our studies together provide strong evidence that the Kv2.1-syntaxin interaction exerts its effect on secretion after ATP-dependent priming of the vesicles. Thus, biochemical analysis showing that Ca^2+^ -triggering of ATP-primed cells enhanced coimmunoprecipitation of syntaxin with endogenous Kv2.1 [21] was complemented by functional analysis showing that introduction of the Kv2.1 syntaxin-binding peptides during the Ca^2+^ -triggering step only, was sufficient to exert inhibition of release (Fig. 3).

The Kv2.1 channel is commonly expressed in the soma and dendrites of neurons [32]–[36] where it could influence the DCV-mediated release of neuropeptides (e.g. dynorphin and vasopressin) and neurotrophins, and in neuroendocrine cells [18]–[20] where it is well positioned and shown to regulate DCV-mediated hormone release. Together with the indirect effect of Kv2.1 to inhibit secretion by repolarizing the membrane potential and limiting Ca^2+^ influx through voltage-gated Ca^2+^ channels, the apparently antagonistic direct Kv2.1 effect to enhance release contributes to effective DCV release which occurs in response to repetitive firing.

## Materials and Methods

### Constructs and Antibodies

The primary antibodies used were anti Kv1.1-C terminus and Kv2.1-C terminus (Alomone Labs Jerusalem, Israel), monoclonal anti HPC-1 (Sigma Israel, Rehovot), monoclonal anti SNAP-25 (BD Transduction Lab, Lexington, KY),

The DNAs of Kv1.1 and Kv2.1 fragments for production of GST fusion proteins were constructed as described previously ([37], [38]. The fusion proteins were synthesized as described in [38].

### Cracked PC12 cell assay

PC12 cells were grown as described earlier[21]. For the cracked cells assay, cells were labeled and treated as described elsewhere [22], [24], [25]. Briefly, cells were labeled overnight with [^3^H]norepinephrine (NE) (Amersham Corp., Arlington Heights, IL) in the presence of 0.5 mM sodium ascorbate. Unincorporated [^3^H]NE was removed The cells were harvested from the flask with ice-cold KGlu buffer (20 mM Hepes, pH 7.2, 120 mM potassium glutamate, 20 mM potassium acetate, 2 mM EGTA and 0.1% BSA). The cells were permeabilized by a single passage through a specific PC12 designed homogenizer [22], [24], [25] and were incubated with EGTA to fully extract soluble components. Then the cells were resuspended in KGlu buffer with or without a GST-fused protein (final concentration as indicated in the figure legends). Cells were then primed and and triggered in the presence of 1.6 mM CaCl_2_ (1 µM free ionic [Ca^2+^];[39]). After pelleting the cells,the level of release was calculated as a percentage of the counts in the supernatant out of the total counts in both pellet and supernatant.

### Pull-down of PC12 cell proteins

GST fusion proteins (150 pmol) immobilized on glutathione–Sepharose beads were incubated with 10^6^ PC12 cells in KGlu buffer (50 mM HEPES (pH 7.2), 105 mM Potassium glutamate, 20 mM potassium acetate, 2 mM EGTA) with 1% CHAPS and a mixture of protease inhibitors (Boehringer Mannheim) at 4°C for 12 hr. Samples were washed 3 times with KGlu containing 0.1% CHAPS, boiled for 10 min in SDS sample buffer, electrophoresed (12% polyacrylamide gel) and immunoblotted with monoclonal antibody against syntaxin 1A (HPC-1).

### Immunoprecipitation of PC12 cell proteins

5×10^6^–1.5×10^7^ Cells were suspended in lysis buffer (20 mM Tris, pH 7.5, 5 mM EDTA, 100 mM EGTA, 100 mM NaCl, and 1% Triton X-100), supplemented with protease inhibitor cocktail (Boehringen_Mannheim), incubated for 1 h at 4°C, and centrifuged for 10 min at 4°C at 14,000 rpm. Immunoprecipitation (IP) reactions from cells were carried out in the presence of 1% CHAPS and separated by SDS-PAGE, blotted and detected by antibodies (IB).

### Statistical analysis

Data are presented as means±SEM. One way analysis of variance was used to calculate the statistical significance of differences between several groups

## Supporting Information

Figure S1 Interaction between syntaxin and SNAP-25, VAMP2 or synaptotagmin in PC12 cells is not hampered significantly by a syntaxin-binding peptide. PC12 cells lysates were immunoprecipitated by anti SNAP-25 or by anti syntaxin antibodies (left and right panels, respectively) in the absence (no peptide) or presence of 10 µM Kv2.1-C1a or Kv1.1-C (as control) peptides, as indicated below the lanes. Immunoprecipitation (IP) reactions from 5×10^6^–1.5×10^7^ cells were carried out in the presence of 1% CHAPS and separated by SDS-PAGE, blotted and detected by antibodies (IB) against syntaxin (Syx), SNAP-25, VAMP2 and synaptotagmin (Syt) as indicated. PC12 lysates (6% of pull down reaction) and 10 µg rat brain membranes (RB) were loaded for reference.
(1.80 MB TIF)

Figure S2 Comparison of [3H]-NE Release (in the absence of peptides) in the presence or absence of MgATP in the triggering reaction; the latter was 92% of the former.
(0.34 MB TIF)

Text S1 Supplementary information
(0.03 MB DOC)

Text S2 Supporting information
(0.02 MB DOC)
