# Supplementary figures and images for: Direct Interaction of Endogenous Kv Channels with Syntaxin Enhances Exocytosis by Neuroendocrine Cells
Source: PLoS One. 2008 Jan 2;3(1):e1381. doi: 10.1371/journal.pone.0001381 (PMC2148073; doi:10.1371/journal.pone.0001381)

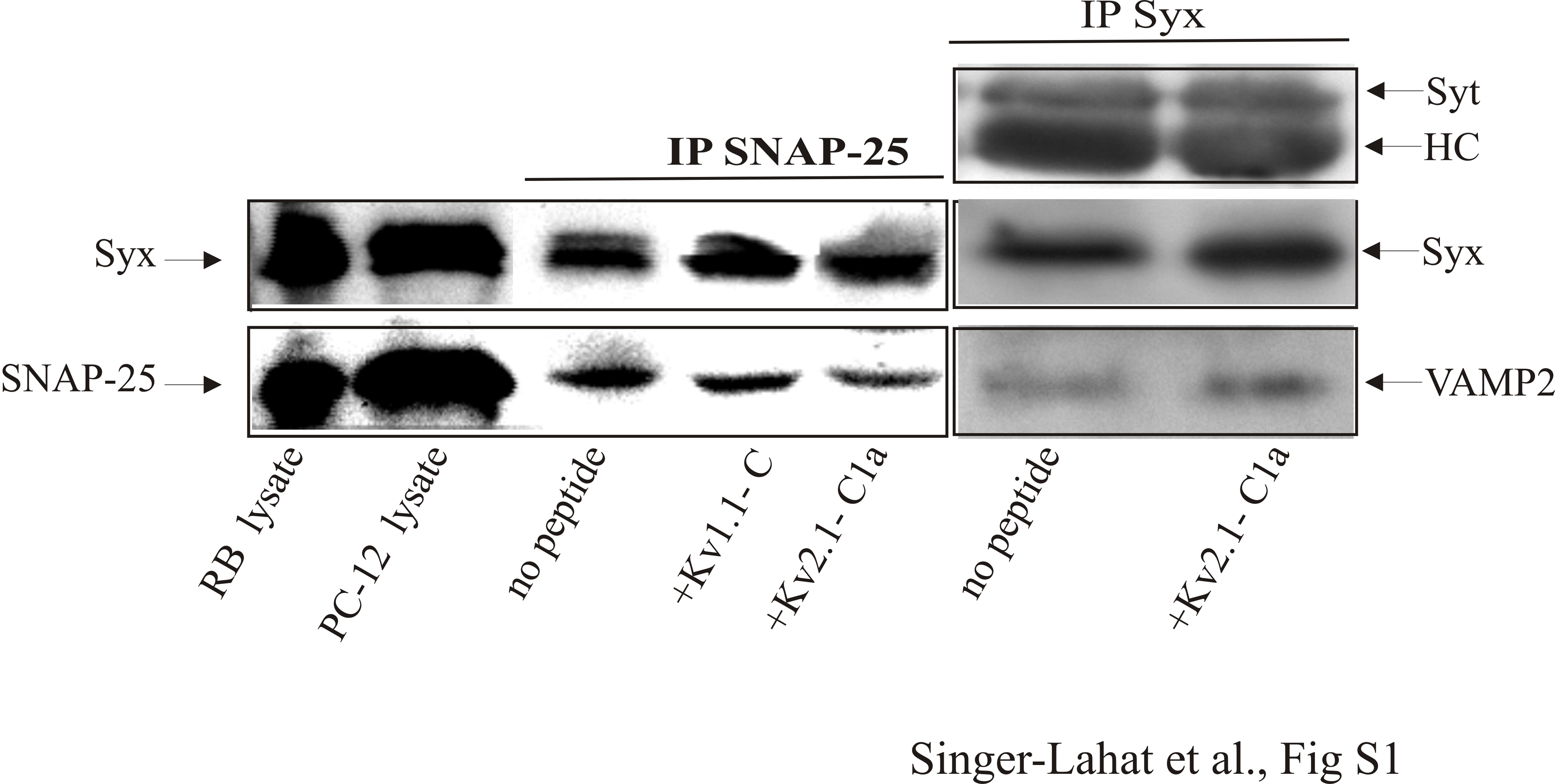

Supplement: Figure S1 — Interaction between syntaxin and SNAP-25, VAMP2 or synaptotagmin in PC12 cells is not hampered significantly by a syntaxin-binding peptide. PC12 cells lysates were immunoprecipitated by anti SNAP-25 or by anti syntaxin antibodies (left and right panels, respectively) in the absence (no peptide) or presence of 10 µM Kv2.1-C1a or Kv1.1-C (as control) peptides, as indicated below the lanes. Immunoprecipitation (IP) reactions from 5×10^6^–1.5×10^7^ cells were carried out in the presence of 1% CHAPS and separated by SDS-PAGE, blotted and detected by antibodies (IB) against syntaxin (Syx), SNAP-25, VAMP2 and synaptotagmin (Syt) as indicated. PC12 lysates (6% of pull down reaction) and 10 µg rat brain membranes (RB) were loaded for reference. (1.80 MB TIF) [file pone.0001381.s001.tif]
