# Supplementary material for: Direct Interaction of Endogenous Kv Channels with Syntaxin Enhances Exocytosis by Neuroendocrine Cells
Source: PLoS One. 2008 Jan 2;3(1):e1381. doi: 10.1371/journal.pone.0001381 (PMC2148073; doi:10.1371/journal.pone.0001381)
Supplement: Text S1 — Supplementary information (0.03 MB DOC) [file pone.0001381.s003.doc]

**Text S1**

***The effect of Kv2.1 syntaxin-binding peptides on the interaction of syntaxin with the SNARE proteins and synaptotagmin.***

Since the Kv2.1 syntaxin-binding peptides had an inhibitory impact on secretion we decided that in order to link their action primarily to the impairment of the channel-syntaxin interaction, it was necessary to ascertain that the peptides did not significantly impair the interaction of syntaxin with its partners in the fusion complex SNAP-25, VAMP2 and synaptotagmin. Such impairments would impact on release without necessarily involving disruption of the syntaxin-Kv2.1 interaction. Thus, in a co-immunoprecipitation experiment, using antibody directed at SNAP-25, the amount of syntaxin that co-immunoprecipitated with SNAP-25 in the presence of 10 μM Kv2.1-C1a peptide (*+Kv2.1-C1a*) did not differ significantly from that observed in the absence of the peptide (*no peptide*) or in the presence of 10 μM Kv1.1-C peptide that does not bind syntaxin (*+Kv1.1-C*) (Fig. S1; left panel). Further, in a co-immunoprecipitation experiment, using antibody against syntaxin, the Kv2.1-C1a peptide did not significantly affect the amount of VAMP2 that co-immunoprecipitated with syntaxin; the amount of co-precipitated synaptotagmin was reduced by ~15% in the presence of the peptide. Importantly, under similar experimental conditions the same peptide concentration considerably reduced (about 90%) the amount of syntaxin that was co-immunoprecipitated with Kv2.1 (Singer-Lahat et al., 2007)

**Reference**

1. Singer-Lahat, D., Sheinin, A., Chikvashvili, D., Tsuk, S., Greitzer, D., Friedrich, R.,Feinshreiber, L., Ashery, U., Benveniste, M., Levitan, E.S. and Lotan, I. (2007) K+ channel facilitation of exocytosis by dynamic interaction with syntaxin*. J Neuros*ci**,** 27, 1651-1658.
