# Supplementary material for: Direct Interaction of Endogenous Kv Channels with Syntaxin Enhances Exocytosis by Neuroendocrine Cells
Source: PLoS One. 2008 Jan 2;3(1):e1381. doi: 10.1371/journal.pone.0001381 (PMC2148073; doi:10.1371/journal.pone.0001381)
Supplement: Text S2 — Supporting information (0.02 MB DOC) [file pone.0001381.s004.doc]

**Text S2**

***Presence of MgATP in the triggering reaction increases release.***

We suspected that the greater effect apparent in the second setting was due to the absence of MgATP during the triggering reaction. Thus, we compared experiments, performed in the absence of peptides, with and without MgATP present in the triggering reaction and found that omission of MgATP reduced release by about 8% (Fig. S2), as expected.
